# Genetic polymorphism data support a relationship between schizophrenia and microsatellite variability in *PLA2G4A* in Northern Europeans not Han Chinese

**DOI:** 10.1002/ajmg.b.32881

**Published:** 2021-12-09

**Authors:** Craig J. Hudson, Adam M. R. Groh, Fabio Macciardi, Rhys C. Hudson

**Affiliations:** ^1^ Biosential Inc. Toronto Ontario Canada; ^2^ Montreal Neurological Institute‐Hospital McGill University Montréal Québec Canada; ^3^ Department of Psychiatry University of California, Irvine (UCI) Irvine California USA; ^4^ Concordia University Montréal Québec Canada

A recent reanalysis of two studies of *PLA2G4A* microsatellite polymorphism in schizophrenia found a positive association when length of the microsatellite was considered (Hudson, Zhu, & Durocher, 2021). These now positive results from samples from Edinburgh, Scotland, and Pittsburgh, United States, are consistent with a study of Canadian and Northern Italian families (Hudson et al., 1996). These positive findings stand in contrast to negative results (Frieboes et al., 2001) utilizing the same microsatellite to investigate differences between 300 patients with schizophrenia and 300 matched healthy controls. The length of the microsatellite was not considered in the sample that was composed of an even mixture of subjects from German and Han Chinese descent. This data set could not be combined with the original reanalysis as the absolute length of the polyA sequence was not given. Length can be inferred, however, from allele number that correlates with polyA length allowing for a within study reevaluation of this variable as well as the potential role of ethnicity.

Functional aspects of microsatellites were not known at the time of the original *PLA2G4A* microsatellite polymorphism in schizophrenia studies and the marker was utilized only for its polymorphic attributes (Chowdari et al., 2001; Frieboes et al., 2001; Hudson et al., 1996; Price, Fox, St Clair, & Shaw, 1997). It is now recognized that length microsatellites can contribute to neurodevelopmental disorders such as schizophrenia by impairing the expression of *PLA2G4A* through alteration of nucleosome position (Bagshaw, 2017) or by causing replication errors that result in the production of a dysfunctional or improperly regulated cPLA_2_ enzyme (Leclercq, Rivals, & Jarne, 2010). The proximity of the polyA marker (1Kb upstream from the promoter region) suggests a functional role for this polyA sequence in the etiopathogenesis of central nervous system (CNS) illnesses that implicate the cPLA_2_ enzyme (Farooqui, Ong, Horrocks, & Farooqui, 2000). Importantly, recent work has uncovered a significant number of noncoding RNA elements that regulate the expression of *PLA2G4A* and are differentially expressed in the dorso‐lateral prefrontal cortex (DLPFC) between people diagnosed with schizophrenia and controls subjects. These elements are putatively acting as weak enhancers, ultimately leading to an overexpression of the *PLA2G4A* transcripts in schizophrenia (Guffanti et al., 2018). Therefore, considering the potential regulatory role of the length microsatellite, and the recent reanalysis that supports a relationship between length of the polyA microsatellite and schizophrenia in other samples, a reevaluation of the Frieboes et al.'s data set is indicated. Additionally, it is noted that subjects with schizophrenia in previously studied Canadian and Italian samples (Hudson et al., 1996) are significantly more likely to have both alleles in the longer polyA range (χ^2^ test, *p* < .005). Furthermore, in this past study, the schizophrenia group was separated into two sets based on nicotinic acid sensitivity, a putative measure of cPLA_2_ dysfunction at that time (Hudson, Lin, Cogan, Cashman, & Warsh, 1997) but now validated (Yao et al., 2016). The nicotinic acid‐insensitive patients displayed longer allelic variants than the nicotinic acid‐sensitive patients.

Here, we revisit the Frieboes et al.'s data set and analyze by allele length also, separated by ethnicity: Northern European (Germany) and Han Chinese (Taiwan, China) descent. These groups were distinguished at the time of the original study but only the Han Chinese sample was analyzed separately. Analysis of the overall pooled sample of 300 patients diagnosed with schizophrenia and 300 matched controls found no association with the extreme length of microsatellite and schizophrenia (*p* = .2514; odds ratio [OR] = 1.242; 95% confidence interval [CI], 0.7212–2.107). Similarly, a separate analysis of the Chinese sample also found no evidence of linkage of extreme length with schizophrenia (*p* = .3629; OR = 0.8308; 95% CI, 0.4224–1.651). A 2 × 2 Chi‐square analysis with patients diagnosed with schizophrenia versus controls and normal versus extreme length polyA, however, now finds significantly greater frequency of extreme polyA alleles in the group diagnosed with schizophrenia in the population of Northern European descent (*p* = .0465; OR = 2.269; 95% CI, 1.000–5.161). Figure 1 plots allele frequency data from isolated Han Chinese (a) and Northern European (b) samples.

**FIGURE 1:**
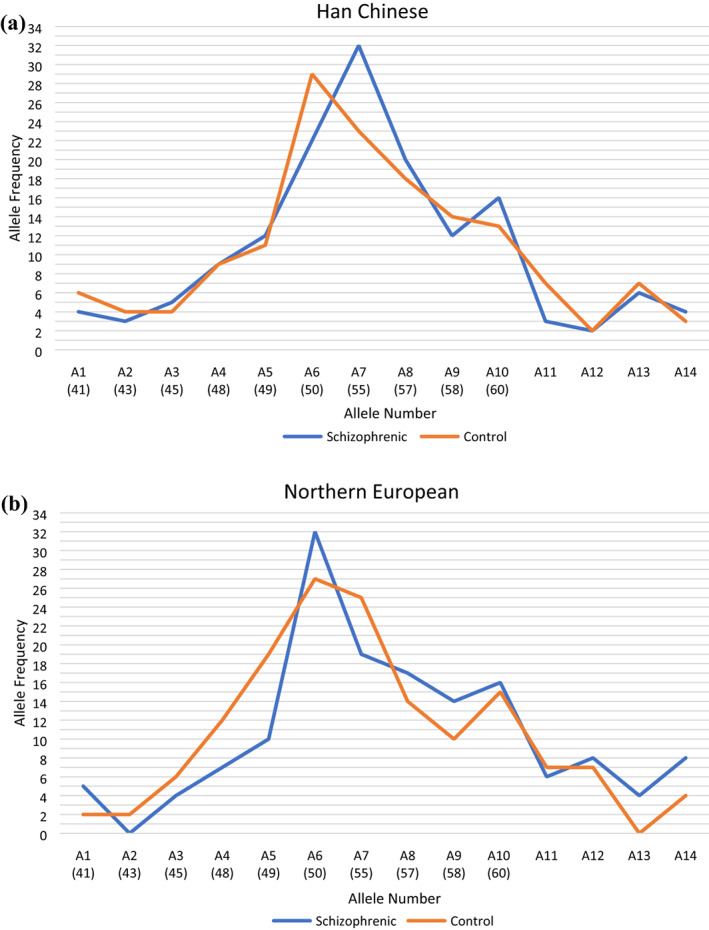
Allele frequency data from isolated Han Chinese (a) and Northern European (b) samples. Fourteen different lengths of the polyA microsatellite are represented on the x‐axis (A1‐A14) for both populations, both of which were isolated from the total sample from Frieboes et al. (2001)

This analysis provides further evidence that the polyA microsatellite near the promoter region of the *PLA2G4A* gene may serve as a functional marker for schizophrenia. This linkage of the larger microsatellite region of this marker is now found in all samples from Northern European descent (a total of 348 patients and 346 controls) when analyzed by length. All published studies of this marker demonstrate a preponderance of extreme polyA sequences in people suffering from schizophrenia except for one sample from the United Kingdom with a small sample size and unclear number of alleles derived from controls (Doris, 1998).

While the possibility of impaired cPLA_2_ activity in schizophrenia exists, a clear consensus about this does not exist with different groups identifying higher, lower, or no difference in cPLA_2_ activity in blood samples of patients diagnosed with schizophrenia compared with samples from healthy controls (Law, Cotton, & Berger, 2006; see meta‐analysis in Xu et al., 2019). These discrepancies likely arise from difficulties with correlating serum, plasma, or platelet PLA_2_ activity with the activity of cPLA_2_ in the brain as PLA_2_ activity in blood samples can emerge from either cPLA_2_ activity or other forms of PLA_2_. Furthermore, even studies that differentiated between cPLA_2_ and other forms of PLA_2_ did not measure cPLA_2_ activity or the amount of protein produced from the *PLA2G4A* gene specifically. Analysis of cPLA_2_ activity in post‐mortem brain tissue revealed decreased activity in specific brain regions of patients with schizophrenia (Ross, Turenne, Moszczynska, Warsh, & Kish, 1999). Similar to the analysis of cPLA_2_ activity in the blood, the identification of which gene product is responsible for the differences remains unknown.

Consequently, we argue that an association between potentially decreased CNS cPLA_2_ activity and schizophrenia pathophysiology needs additional study. In particular, a loss‐of‐function or decreased function of *PLA2G4A* would explain a vulnerability to neurodevelopmental disorders such as schizophrenia. It is, therefore, important to revisit investigation of this potential genetic predisposition for schizophrenia and the mechanistic consequences of this specific microsatellite thereof.

## CONFLICTS OF INTEREST

Dr. C. J. Hudson holds several patents pertaining to Central Nervous System Signal Transduction.

## Data Availability

The data that support the findings of this study are available in: doi.org/10.1002/ajmg.1262.
